# Severe Acral Necrosis Complicating Thrombotic Microangiopathy in Two Toddlers

**DOI:** 10.1155/2020/8869883

**Published:** 2020-12-15

**Authors:** H. Nassih, Z. Lazrak, S. Younous

**Affiliations:** ^1^From B Pediatric Ward, Department of Pediatrics, Child and Mother Hospital, Mohammed VI University Hospital Center, Marrakesh Medical and Pharmacy Faculty, Caddy Ayad University, Marrakesh, Morocco; ^2^From the Pediatric Intensive Care Unit, Department of Pediatrics, Child and Mother Hospital, Mohammed VI University Hospital Center, Marrakesh Medical and Pharmacy Faculty, Caddy Ayad University, Marrakesh, Morocco

## Abstract

Acral ischemia/necrosis is one of the rarest but most dreadful complications of thrombotic microangiopathy in pediatric patients. It is more reported with thrombotic thrombocytopenic purpura than with hemolytic and uremic syndrome. Even with anticoagulant therapy, it is often irreversible, leading to amputation.

## 1. Introduction

Thrombotic microangiopathy (TM) leads to microcirculatory thrombosis. Ischemic changes that follow are more frequently seen in the central nervous and renal systems. Musculoskeletal system involvement is less described. However, when it occurs, acral necrosis is often severe. We report two toddlers presenting with an acute onset of TM complicated by bilateral acral ischemia.

## 2. Case Report

### 2.1. Case One

An eleven-month-old and first-degree consanguineous boy was admitted to the pediatric intensive care unit (PICU) for acute neurological distress. He was treated for pneumonia ten days ago and had taken oral amoxicillin 100 mg/kg/day. His antipneumococcal vaccination was up to date. The mother reported rare-black urines during the last three days. The last 24 hours before admission, the child was drowsy and hypotonic. At admission to the pediatric emergency department, first clinical evaluation found a comatose child with a Glasgow score of 8/15. He had generalized edema, pallor, and dyspnea. He had normal body temperature of 36.5°C. His pulseoximetry found SpO_2_ of 75%. He also had hypertension of 167/87 mmHg. After intubation and mechanical ventilation, chest radiograph was performed and was normal. Blood, urine, stool, and bronchial samples were collected for bacteriological tests. He was oliguric at 0.3 cc/kg/h. Intravenous furosemide was given at 6 mg/kg/day. Because of the onset of coma, pallor, and oliguria after an episode of upper respiratory tract infection, thrombotic thrombocytopenic purpura (TTP) was highly suspected. Blood tests found severe hemolytic anemia (hemoglobin of 4 g/dl, haptoglobin of 0.01 g/l, reticulocytes of 134000/mm^3^, LDH of 2390 UI/l, and ferritin of 985 ųg/ml), thrombocytopenia (platelets of 79000/mm^3^), acute renal failure, hyperkalemia, and acidosis (urea of 2.04 g/l, creatinine of 19 mg/l, K^+^ of 7 mmol/l, HCO^3−^of 8 mmol/l, and pH of 7.1). Meanwhile, his CRP and ESR were normal (respectively, 2 mg/l and 6 mm the first hour). After catheter placement, continuous peritoneal dialysis was started. The child was transfused with blood and put under nicardipine per IV. Bronchial, blood, and stool samples were sterile. On the second day of his admission to the PICU, the child was still anuric with less than 1 cc/kg/day of urine output.

**Figure 1:**
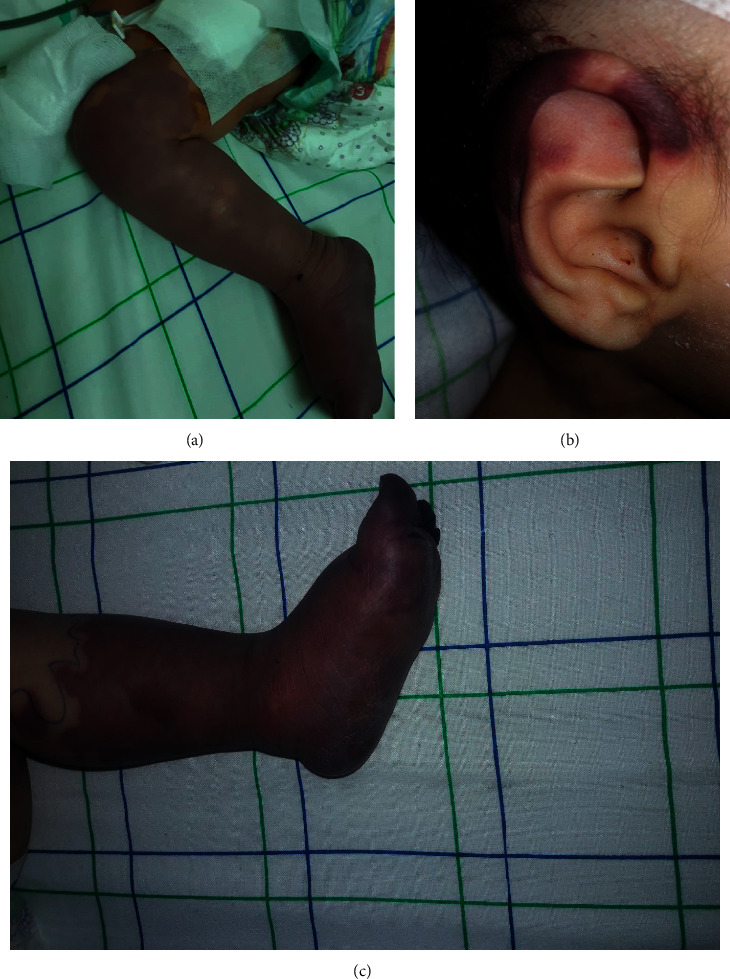
(a) An eleven-month-old boy with acral necrosis of the right foot. (b, c) A seven-month-old boy with distal necrosis of the foot and ears.

Meanwhile, he developed acral ischemia of the right leg, which was rapidly extensive within a few hours (Figure 1(a)). The Doppler sonography found a thrombus of the right external iliac artery. Permeabilization surgery was performed, and intravenous unfractionated heparin (initially 75 UI/kg, then by continuous intravenous infusion at 20 UI/kg/1 h) was started. On the third day of hospitalization, the ischemia extended to the left leg. The thrombophilia workup was unremarkable (PTT, aPTT, INR, fibrinogen, D-Dimers, antithrombin III, and protein C/S), as well as the C3/C4 complement fractions. Unfortunately, hemodynamic instability and neurological deterioration kept worsening. We lost the child after three days of PICU hospitalization.

### 2.2. Case Two

A seven-month-old boy was admitted to PICU for acute respiratory distress. Fifteen days ago, he had acute bloody diarrhea and fever of 40°C. His doctor suspected dysentery of bacterial origin and prescribed oral antibiotics (sulfamethoxazole-trimethoprim) for five days. Afterwards, the boy regained apyrexia, and the stool was back to normal. But, by the tenth day, the child developed generalized edema, gross hematuria, and oliguria. He was admitted to the pediatric emergency department, and first evaluation found a conscious child, with a Glasgow score of 13/15. He had severe respiratory distress, with hypoxia (respiratory rate of 65 cycles/min, and SpO2 of 51%) and pallor. He was apyretic at 37°C and had hypertension of 154/89 mmHg. Chest radiograph found signs of acute pulmonary edema. He was anuric even after ureteral catherization and an intravenous bolus of 5 mg/kg of furosemide. The respiratory state kept worsening despite noninvasive ventilation, so the child was intubated. Laboratory workup revealed severe acidosis (HCO^3−^ of 5 mmol/l and pH of 7), acute renal failure (urea of 1.9 g/l, creatinine of 18.4 mg/l), hyponatremia, and hyperkalemia (K^+^ of 5.6 mmol/l and Na^+^ of 125 mmol/l). Normochromic-normocytic-anemia (hemoglobin of 5.5 g/dl) and thrombocytopenia (platelets of 36000/mm^3^) were found on complete cell blood count. Meanwhile, CRP and ESR were normal. Haptoglobin was indetectable, and reticulocyte count, LDH, and ferritin levels were very high (respectively, 165200/mm^3^, 1450 UI/l, and 735 ųg/ml), pointing towards hemolytic and uremic syndrome (HUS). Complement fractions (C3/C4/CH50) were within normal range. Peritoneal dialysis was started, along with intravenous nicardipine. Blood and platelets transfusion were performed. Coprology and PRC of stool were negative. At the third day of PICU stay, we noticed rapidly extensive bilateral acral ischemia of the feet, hands, and ears (Figures 1(b) and 1(c)). The child was put on continuous intravenous unfractionated heparin. After twenty-four hours of starting heparin therapy, ischemia was still extensive and had reached the two knees. The thrombophilia workup was normal. After seven days of the PICU stay, the child died because of persistent hemodynamic instability despite adequate management.

## 3. Discussion

TM, which broadly includes TTP and HUS, is a multisystemic disorder characterized by thrombocytopenia, microangiopathic hemolytic anemia, and ischemic manifestations resulting from platelet agglutination in the arterial microvasculature [1, 2]. Acral necrosis (distal necrosis of fingers and toes), also called peripheral digit ischemic syndrome (PDIS), usually occurs as a sequel to severe Raynaud's phenomenon, a vasospastic disorder frequently related to endothelial cell dysfunction [3]. It is uncommon in pediatric TM [4]; its physiopathology is also unclear [5]. One study reported that, among 94 adult patients with TM, PDIS developed in six of them, and in all these patients, PDIS occurred with postoperative TTP. There were neither arterial nor venous microthrombi observed in any of these patients [6]. The first case described was our eleven-month-old boy. We found no thrombophilia disorder in these children. The lack of facility made MA workup (ADAMST 13 activity, antifactor H antibodies, genetic screening, etc.) impossible. Management of PDIS during HUS-TTP is challenging because of the rapidly extensive course of distal necrosis [2]. Also, initiation of anticoagulation, especially heparin, should be prudent, mainly because of the underlying thrombocytopenia and acute renal failure [7, 8]. Unfortunately, our two cases had a bad prognosis because of the rapidly extensive ischemia and poor neurological and renal outcomes.

## 4. Conclusion

Acral necrosis during MA is rare. A few pediatric cases were reported. Its prevalence increases after surgical and vascular interventions in genetically predisposed individuals. It is more frequent in the course of TTP than HUS. Management is challenging, and distal amputation is often necessary.
